# A Novel Floating High-Voltage Level Shifter with Pre-Storage Technique

**DOI:** 10.3390/s22051774

**Published:** 2022-02-24

**Authors:** Qiang Li, Yuan Yang, Haohao Ma, Yangle Zhou, Guolong You, Minmin Zhang, Wei Xiang

**Affiliations:** 1 Department of Automation and Information Engineering, Xi’an University of Technology, Xi’an 710048, China; lq@bttc.edu.cn (Q.L.); mahaohao@stu.xaut.edu.cn (H.M.); yanglezhou@stu.xaut.edu.cn (Y.Z.); youguolong@stu.xaut.edu.cn (G.Y.); minminzhang@stu.xaut.edu.cn (M.Z.); 2 School of Physical Science and Technology, Baotou Teachers’ College, Baotou 014030, China; 3 School of Engineering and Mathematical Sciences, La Trobe University, Melbourne, VIC 3086, Australia; w.xiang@latrobe.edu.au; 4 College of Science and Engineering, James Cook University, Cairns, QLD 4878, Australia

**Keywords:** floating high-voltage level shifter, low propagation delay, pre-storage, regulated strength, pseudosymmetry, HVCMOS

## Abstract

This paper proposes a novel floating high-voltage level shifter (FHV-LS) with the pre-storage technique for high speed and low deviation in propagation delay. With this technology, the transmission paths from input to output are optimized, and thus the propagation delay of the proposed FHV-LS is reduced to as low as the sub-nanosecond scale. To further reduce the propagation delay, a pull-up network with regulated strength is introduced to reduce the fall time, which is a crucial part of the propagation delay. In addition, a pseudosymmetrical input pair is used to improve the symmetry of FHV-LS structurally to balance between the rising and falling propagation delays. Moreover, a start-up circuit is developed to initialize the output state of FHV-LS during the $V_{\mathrm{DDH}}$ power up. The proposed FHV-LS is implemented using 0.3-µm HVCMOS technology. Post-layout simulation shows that the propagation delays and energy per transition of the proposed FHV-LS are 384 ps and 77.7 pJ @$V_{H}$ = 5 V, respectively. Finally, the 500-points Monte Carlo are performed to verify the performance and the stability.

## 1. Introduction

With the increase in power density and switching speed of the power devices, gate drivers with low propagation delays and high operating frequencies are in high demand [1,2,3]. In the application of feedback-based regulation, the gate drivers are developed to operate in the closed-loop mode to respond to the change of loads [4,5,6]. Moreover, some gate drivers feature functions of over-current protection and fault indication [7,8]. For precise regulation and fast protection, low propagation delay is critical for gate drivers. In commercial applications, the propagation delay of state-of-the-art gate drivers is in the order of ten nanoseconds, excluding the delay induced by bonding wires and pads. In gate drivers, multi-voltage power supply technology is applied to balance between switching speed and power consumption [9,10]. The digital units implementing the logic processing are supplied by low voltage to reduce power dissipation, while the circuits composing the output stage are powered by high voltage to carry enough energy for loads. For communication between circuits supplied with different voltage, the level shifter becomes a critical interface in the gate driver [11,12]. In the medium/high-voltage gate driver or half-bridge gate driver, FHV-LS can transfer the control signal from low-side power rail to high-side power rail with floating-ground, sending commands to the output of gate driver. For the high-speed gate driver, the FHV-LS with nanoseconds of propagation delay will have a negative impact on the total propagation delay in gate driver. Therefore, the reduction in propagation delay of FHV-LS will be an essential destination for high-speed gate driver design.

On the one hand, the existing level-shifting circuits have large propagation delays, which are not suitable for high-speed applications. Especially in feedback-based regulation and protection situations, large propagation delay not only affects the regulating accuracy but also suffers from the risk of damage. On the other hand, large propagation delay will lead to major deviation between rising and falling propagation delay. As the operating frequencies increase, the distortions in duty cycle caused by the deviation become more serious. Therefore, this study focuses on the analysis of factors affecting the propagation delay and methods to reduce the propagation delay and the deviation of FHV-LS. The main contributions of our work are outlined as follows:

- A novel level-shifting circuit structure with pre-storage technology is proposed to achieve low propagation delay. Then, the design approach and implementation of the proposed FHV-LS are presented.
- Based on the proposed FHV_LS, the propagation delay is analyzed in detail. Moreover, the factors affecting the propagation delay are revealed for an in-depth insight into the proposed FHV-LS.
- To further improve the performance of the proposed FHV-LS, the pull-up network with regulated strength technology and the pseudosymmetrical input structure are introduced to optimize the propagation delay and the deviation of the proposed FHV-LS, respectively. In addition, a start-up circuit applied to the register is developed to initialize the output state of FHV-LS during $V_{\mathrm{DDH}}$ power-up. With the start-up circuit, the output of FHV-LS always follows the input correctly in any operating condition.

The remainder of this paper is organized as follows. Section 2 reviews the conventional FHV-LSs, and three topologies are summarized. Section 3 presents the design principle and implementation of the proposed FHV-LS and also analyzes the propagation delay. Section 4 proposes three improvements to optimize the propagation delay, deviation, and start-up. The performance of post-simulation is shown in Section 5. Section 6 concludes our work.

## 2. Review of Conventional FHV-LSs

**Figure 1:**
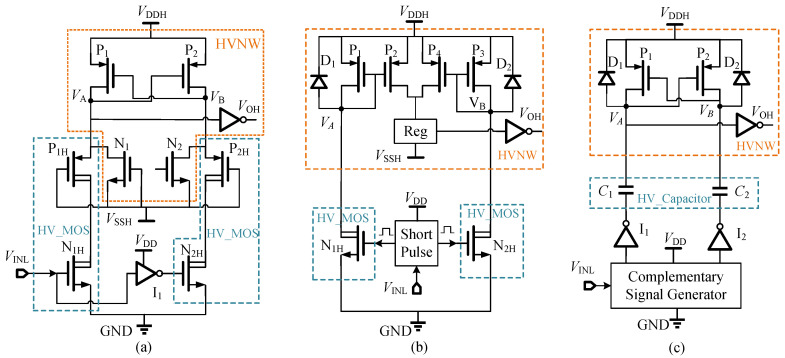
Three topologies of FHV-LS: (**a**) DC-LS, (**b**) CM-LS, (**c**) CC-LS.

Many FHV-LSs have been proposed in the literature [13,14,15,16,17,18,19,20,21,22,23,24,25,26,27,28,29] to reduce the propagation delay. According to the structures, three topologies (Topology-I, Topology-II, and Topology- III) are summarized in Figure 1. The devices in orange dashed boxes are placed in HV-NWell (HVNW). The body of HVNW biased with $V_{\mathrm{SSH}}$ can tolerate high voltage. Consequently, the devices in HVNW could operate in the floating high-voltage domain between $V_{\mathrm{DDH}}$ and $V_{\mathrm{SSH}}$. The devices in blue dashed boxes are HVMOS whose drain source and drain gate can handle high voltages, but its gate source switches in the low voltage range.

(1) Topology-I: DC-LS

The first topology of FHV-LS is direct-coupled level shifter (DC-LS), as depicted in Figure 1a. The HV-NMOS transistors $N_{1H}$/$N_{2H}$ are used to convert the input signal $V_{\mathrm{INL}}$ into its drain current. The HV-PMOS transistors $P_{1H}$/$P_{2H}$ transfer its drain current into voltage and isolate the high drain voltage to protect the devices in HVNW. The NMOS N${}_{1}$/N${}_{2}$ are used to clamp the voltage $V_{A}$/$V_{B}$ to not fall below $V_{\mathrm{SSH}}$. The DC-LS in [13,14] is a basic structure of FHV-LSs which features a sample structure and fewer components. The circuits are fabricated using silicon-on-insulator (SOI) technology to improve the voltage rate, but it has a large propagation delay and poor symmetry between the rising and falling propagation delays. The asymmetry between rising and falling propagation delay will induce deviation, which will increase with the decrease of propagation delay. To reduce the static power consumption, the input of FHV-LS is activated by a short pulse generator [15,16], but the propagation delays are still unsatisfactory, and the deviation between rising and falling propagation delay in [15] is not optimized. The DC-LSs proposed in [17,18] decrease the propagation delay and optimize the symmetry between the rising and falling propagation delays; however, the propagation delay is at least 2 ns. The nanosecond delay may not meet the needs of high-speed applications.

(2) Topology-II: CM-LS

The second topology of FHV-LS structure is the current-mirror level shifter (CM-LS), as shown in Figure 1b. Compared with DC-LS, CM-LS has a large quiescent current induced by the current mirror. To improve the power efficiency, the HVNMOS transistors $N_{1H}$/$N_{2H}$ of CM-LS are controlled by pulse signals. Reg is a register to hold the output state. The current mirrors, composed of $P_{1}$/$P_{2}$ and $P_{3}$/$P_{4}$, are used to change the state of Reg according to the input state of $V_{\mathrm{INL}}$. The diodes D${}_{1}$/D${}_{2}$ are reversely paralleled with $P_{1}$/$P_{3}$ to clamp the voltage $V_{A}$/$V_{B}$ to protect the devices in HVNW. The CM-LS in [19,20,21] utilizes a short pulse generator to control HV-MOS $N_{1H}$/$N_{2H}$. With signal conversion, the state of the latch unit is changed by the short pulse current. To reduce power consumption, an instantaneous-power-consuming level shifter is proposed to increase the efficiency [22] since the output detector is used to turn the level shifter off before the delay time. The CM-LSs proposed in [23,24,25] alter the state of RS-trigger by the voltage cross a resistor. This structure is commonly found in higher-voltage level shifters. It also needs the Schottky diodes in reverse parallel across the resistors to protect the devices in HVNW. Considering the influence of the process, temperature, and parasitic effect, the pulse width of the short pulse should have enough margins to guarantee that the latch unit can be flipped reliably. However, extending the width of the input pulse will lead to an increase in the power consumption and design complexity. Therefore, the pulse width should be optimized according to the power consumption and performance.

(3) Topology-III: CC-LS

The third topology of FHV-LS is a capacitor-coupled level shifter (CC-LS), as shown in Figure 1c. High-voltage capacitors, $C_{1}$ and $C_{2}$, are used to shift the input signal $V_{\mathrm{INL}}$ to the output $V_{\mathrm{OH}}$ with the high common-mode voltage. The CC-LS proposed in [26] has a propagation delay of 0.5 ns and low static current consumption, but requires an off-chip capacitor to enhance the power PMOS driving capability. The CC-LS in [27] integrates two on-chip capacitors (both 60 fF) to shift the signal from $V_{\mathrm{INL}}$ to $V_{\mathrm{OH}}$, but the rising/falling propagation delay of 1.45 ns/1.3 ns is large for the 180 nm process. The CC-LS in [28] achieves a 0.5 ns propagation delay with two 2 pF on-chip capacitors. The CC-LS in [29] achieves a 115 ps propagation delay by isolated low-voltage NMOS transistors and capacitive coupling, but the current mirror of the level shifter in [28,29] is not turned off after converting, resulting in high power consumption.

## 3. Proposed FHV-LS

### 3.1. Design Approach of Proposed FHV-LS

**Figure 2:**
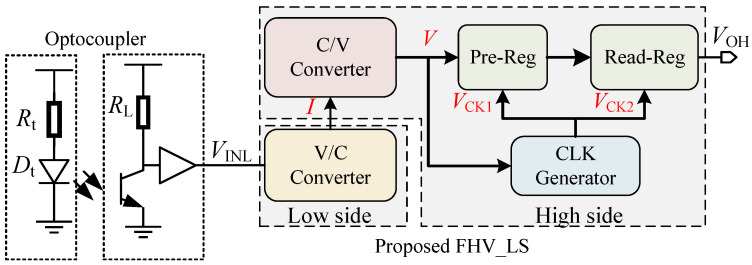
Topology of the proposed FHV-LS.

The gate drivers with FHV-LS are commonly used to control the power devices. However, the electrical noise generated by power circuits is also coupled to the driver stage by means of electricity or magnetism. To reduce the risk of false trigger, optocouplers are applied to achieve electrical isolation between the control signals and the high-voltage signals. Then, the optocoupler transmits the control signal to the input of the level-shifting circuit as shown in Figure 2. The proposed FHV_LS is composed of high-side circuit and low-side circuit. The high-side circuit consists of C/V converter, registers (pre-Reg, read-Reg), and CLK generator. The V/C converter on the low side converts the input voltage into the current signal I. The C/V converter on the high side then transforms the current I into the voltage V which is the input to the CLK generator and pre-Reg. The CLK generator generates the control signals $V_{\mathrm{CK}1}$ and $V_{\mathrm{CK}2}$ to enable the pre-Reg and read-Reg in the latch or transfer mode. If pre-Reg operates in the transfer mode, the output of the C/V converter is stored in pre-Reg, while read-Reg works in the latch mode to hold $V_{\mathrm{OH}}$. Conversely, pre-Reg operates in latch mode, and read-Reg is in transfer mode to transmit the output of pre-Reg to $V_{\mathrm{OH}}$. Based on the mode switching for registers, the "next" state of $V_{\mathrm{INL}}$ is loaded in pre-Reg in the steady state. During the switching of $V_{\mathrm{INL}}$, the input state restored in pre-Reg is transmitted to the output $V_{\mathrm{OH}}$ under the control of $V_{\mathrm{CK}1}$/$V_{\mathrm{CK}2}$. In other words, the present state of $V_{\mathrm{INL}}$ is transmitted to $V_{\mathrm{OH}}$. Therefore, the propagation delay of FHV-LS is determined by the delay from $V_{\mathrm{INL}}$ to $V_{\mathrm{CK}2}$.

### 3.2. Implementation of the Proposed FHV-LS

**Figure 3:**
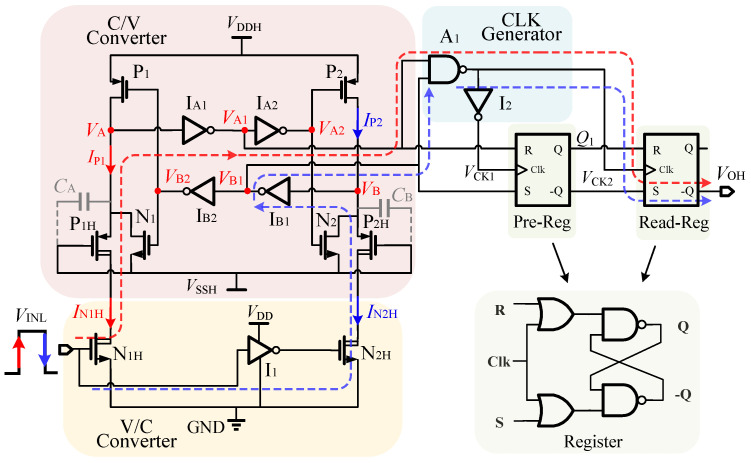
Implementation of the proposed FHV-LS.

The implementation of the proposed FHV-LS is depicted in Figure 3. It involves two power rails, namely the low-side power rail ($V_{\mathrm{DD}}$-GND) and the high-side power rail ($V_{\mathrm{DDH}}$-$V_{\mathrm{SSH}}$). The V/C converter on the low side is composed of HV-NMOS ($N_{1H}$-$N_{2H}$) and inverter $I_{1}$. The logic states of $N_{1H}$ and $N_{2H}$ are flipped by $I_{1}$. The C/V converter on the high side consists of transistors ($P_{1}$-$P_{2}$, $N_{1}$-$N_{2}$), inverters $I_{A1}$-$I_{A2}$, $I_{B1}$-$I_{B2}$), and HV-PMOS ($P_{1H}$-$P_{2H}$). The CLK generator is composed of the Nand gate $A_{1}$ and inverter $I_{2}$. The pre-Reg/read-Reg registers controlled by $V_{\mathrm{CK}1}$/$V_{\mathrm{CK}2}$ are clock-controlled RS flip-flops. The signal transmission paths of proposed FHV-LS from $V_{\mathrm{INL}}$ to $V_{\mathrm{OH}}$ are highlighted with red and blue dashed lines during $V_{\mathrm{INL}}$ witches. To make the formula clear, "0/1" represent logic low/high for the high-side and low-side logic signals.

**Figure 4:**
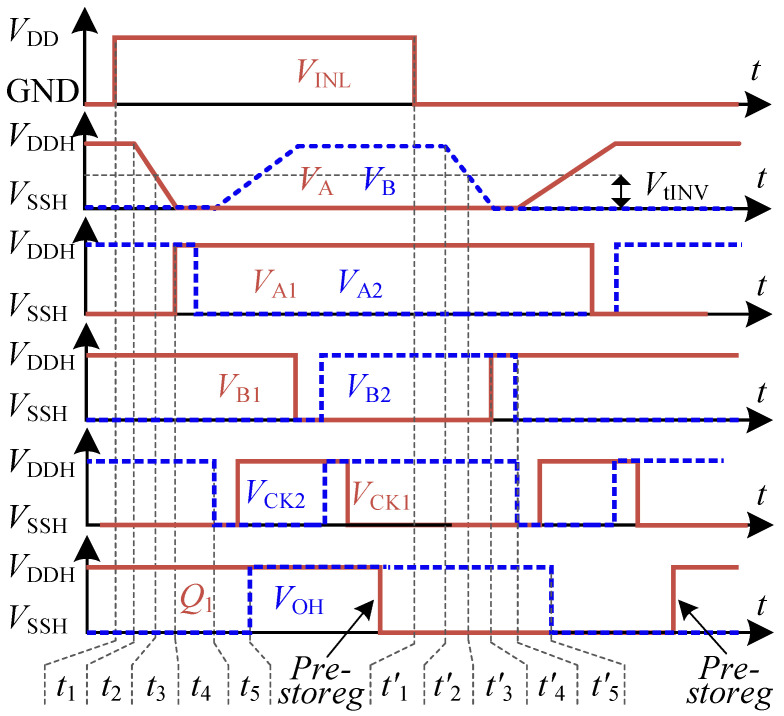
Propagation delay segments of proposed FHV-LS.

The operating process of the proposed FHV_LS is depicted as shown in Figure 4. We assume that $V_{\mathrm{INL}}$ = 0 is the initial input state. In this state, $N_{1H}$, N${}_{1}$, $P_{2H}$, and $P_{2}$ are turned off. $N_{2H}$, N${}_{2}$, $P_{1H}$, and $P_{1}$ are turned on and operate in the linear region. So, $V_{A}$ = $V_{A2}$ = $V_{B1}$ = 1, $V_{A1}$ = $V_{B}$ = $V_{B2}$ = 0. The outputs of the CLK generator are $V_{\mathrm{CK}1}$ = 0 and $V_{\mathrm{CK}2}$ = 1. According to the register, pre-Reg operates in the transfer mode, while read-Reg is in the latch mode. The output of pre-Reg is determined by the logic of $V_{A1}$ and $V_{B1}$, so $Q_{1}$ = 1. It indicates that the next state of $V_{\mathrm{INL}}$ is loaded in pre-Reg in the steady state. For the latch mode of read-Reg, it is worth mentioning that the output of read-Reg is an uncertain value during $V_{\mathrm{DDH}}$ power-up. So, the start-up circuit is required to force the output state of read-Reg to be $V_{\mathrm{OH}}$ = 0. The start-up circuits will be discussed in Section 4.

When $V_{\mathrm{INL}}$ varies from GND to $V_{\mathrm{DD}}$ rapidly, the signals of FHV-LS are transmitted along with the red dashed line. Firstly, $N_{1H}$ is turned on, and operates in saturation. Then $N_{2H}$ is off after the delay of inverter $I_{1}$. To ensure the high-side logic circuits work well, we must succeed in taking $V_{A}$ below the threshold $V_{\mathrm{tINVH}}$ of inverter $I_{A1}$. It requires that the current $I_{N1H}$/$I_{N2H}$ is larger than the current of $P_{1}$/$P_{2}$ before $V_{A}$ falls below $V_{\mathrm{tINVH}}$. Otherwise, the state of high-side circuits can not be flipped. As voltage $V_{A}$ is lower than the threshold of $I_{A1}$, the output states of inverter $I_{A1}$ are reversed. Thus, $V_{A2}$ = 0, and $P_{2}$ is turned on to pull up voltage $V_{B}$. At the beginning, $V_{B}$ is lower than the threshold of inverter $I_{B1}$, $V_{A1}$ = $V_{B1}$ = 1. During this critical time interval, $V_{\mathrm{CK}1}$ = 1 and $V_{\mathrm{CK}2}$ = 0. Accordingly, pre-Reg operates in the latch mode while read-Reg in the transfer mode. The state of $V_{\mathrm{INL}}$ = 1 stored in the pre-Reg is then transferred to the output of read-Reg. Thus, $V_{\mathrm{OH}}$ = $Q_{1}$ = 1. It can be considered that the current input state $V_{\mathrm{INL}}$ = 1 is transmitted to $V_{\mathrm{OH}}$ during this period. As voltage $V_{B}$ rises above the threshold of inverter $I_{B1}$, $V_{B1}$ = 0, $V_{B2}$ = 1. $P_{1}$ is turned off, and $V_{A}$ is clamped to floating ground $V_{\mathrm{SSH}}$ by the conducted NMOS N${}_{1}$. Then, $N_{1H}$ goes into linear region to reduce the drain current $I_{N1H}$. Due to $V_{\mathrm{CK}1}$ = 0, $V_{\mathrm{CK}2}$ = 1, pre-Reg operates in the transfer mode, and the read-Reg works in latch-mode. The inputs of pre-Reg are $V_{A1}$ = 1 and $V_{B1}$ = 0, so $Q_{1}$ = 0. As a result, pre-Reg is loaded with the next state of $V_{\mathrm{INL}}$ again.

When $V_{\mathrm{INL}}$ varies from $V_{\mathrm{DD}}$ to GND rapidly, $N_{1H}$ is turned off and $N_{2H}$ is turned on after the delay of inverter I${}_{1}$. The signal transmission path is shown as the blue dashed line in Figure 3. Due to the symmetrical circuit structures, the transmission paths are similar. It will not be discussed again.

### 3.3. Propagation Delay Analysis

The propagation delay from $V_{\mathrm{INL}}$ to $V_{\mathrm{OH}}$ can be subdivided into five segments. The segments of the rising and falling propagation delays are depicted in Figure 4. To simplify the expression of equation, we take the following abbreviation: $V_{H}$ = $V_{\mathrm{DDH}}$−$V_{\mathrm{SSH}}$ and all voltages on the high side are referenced by $V_{\mathrm{SSH}}$.

Delay $t_{1}$ is the transmission time of the V/C converter from $V_{\mathrm{INL}}$ to the current $I_{N1H}$. It is determined by the intrinsic delay $t_{d\_\mathrm{NH}}$ of $N_{1H}$/$N_{2H}$ which can be minimized using the minimum channel length to decrease the parasitic capacitance. Delay ${t^{{}^{\prime }}}_{1}$ is the transmission time from $V_{\mathrm{INL}}$ to $I_{N2H}$. Due to the delay $t_{d\_\mathrm{INV}}$ of inverter I${}_{1}$, delay ${t^{{}^{\prime }}}_{1}$ is longer than $t_{1}$. Thus, $t_{1}$ and ${t^{{}^{\prime }}}_{1}$ are given by

(1) $$t_{1}=t_{d\_\mathrm{NH}}$$

(2) $$t_{1}^{\prime }=t_{d\_\mathrm{NH}}+t_{d\_\mathrm{INV}}.$$

After delay $t_{1}$ (${t^{{}^{\prime }}}_{1}$), $N_{1H}$ ($N_{2H}$) is turned on. The parasitic capacitor $C_{A}$ ($C_{B}$) will be discharged to pull down the voltage $V_{A}$ ($V_{B}$). Delay $t_{2}$ is the time taken for $V_{A}$ ($V_{B}$) to fall from $V_{H}$ to the threshold voltage $V_{\mathrm{tINVH}}$ of inverter $I_{A1}$ ($I_{B1}$). $t_{2}$ is denoted as the fall time. In this delay interval, $N_{1H}$/$N_{2H}$ operating in the saturation region is taken as a current source. The current $I_{0}$ can be expressed by

(3) $$I_{0}=I_{N1,2H}=\frac{1}{2}\mu_{n}C_{\mathrm{ox}}{\left(\frac{W}{L}\right)}_{N1,2H}{\left(V_{\mathrm{DD}}-V_{\mathrm{tN}}\right)}^{2}.$$

Since $P_{1}$/$P_{2}$ operates in the linear region, it can be treated as a resistance $R_{P}$.

(4) $$R_{P}=\;{\left[\mu_{p}C_{\mathrm{ox}}{\left(\frac{W}{L}\right)}_{P}\left(V_{H}-V_{\mathrm{tp}}\right)\right]}^{-1}$$

where $V_{\mathrm{tN}}$ and $V_{\mathrm{tp}}$ are the threshold voltages of $N_{1H}$($N_{2H}$) and $P_{1}$($P_{2}$), respectively.

**Figure 5:**
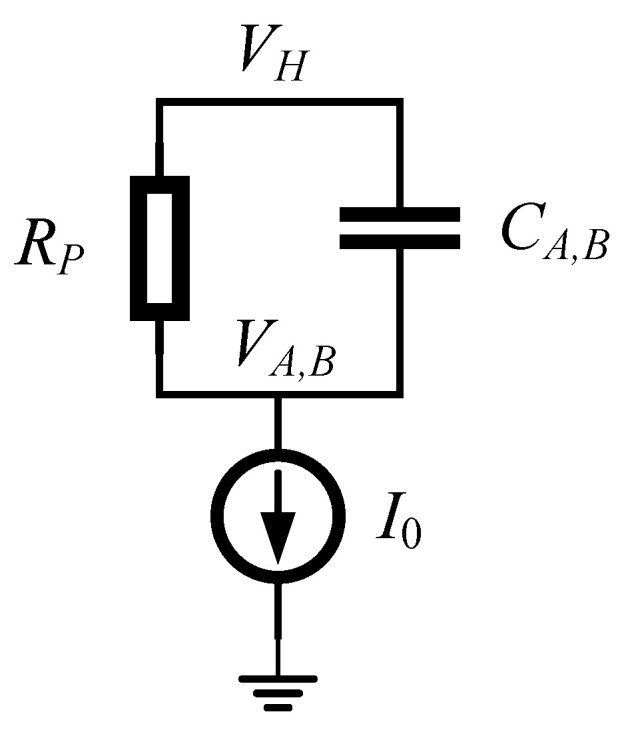
Equivalent circuit during the fall time of $V_{A,B}$.

The equivalent circuit during the fall time of $V_{A,B}$ is shown in Figure 5. The capacitor $C_{A}$ ($C_{B}$) is the total parasitic capacitance at node $V_{A}$ ($V_{B}$). The equivalent circuit can be regarded as an RC network composed of $R_{P}$ and $C_{A}$ ($C_{B}$), which is discharged by the current source $I_{0}$. The RC constant time can be found as $\tau =C_{A,B}R_{P}$. Thus, the voltages can be expressed by

(5) VA,Bt=VH−I0RP1−e−t−tττ.

It is found from Equation (5) that $V_{A,B}\left(t\right)$ will decrease with the increase of *t*. When the time increases to infinity, $V_{A,B}$ will be close to $V_{H}-I_{0}R_{P}$. In other words, $V_{A,B}$ will decrease to a lower value with the increase of $R_{P}$ under the same time condition.

It follows from (5) that the fall time $t_{2}$ (${t^{{}^{\prime }}}_{2}$) can be derived as

(6) $$t_{2}=t_{2}^{\prime }=\tau \ln{\left(1-\frac{V_{H}-V_{\mathrm{tINVH}}}{I_{0}R_{p}}\right)}^{-1}.$$

To flip the state of inverter $I_{A1}$ ($I_{B1}$), $V_{A}$ ($V_{B}$) should fall below the threshold voltage $V_{\mathrm{tINVH}}$ of $I_{A1}$ ($I_{B1}$). Therefore, the relationship should be satisfied in circuit design

(7) $$I_{0}R_{p}>V_{H}-V_{\mathrm{tINVH}}.$$

Usually, $\mu_{n}=2\mu_{p}$ and $|V_{\mathrm{tN}}\left|\approx \right|V_{\mathrm{tp}}|$. Under the typical condition, we have $V_{\mathrm{DD}}$ = $V_{H}$. Plugging (3) and (4) into (7), the sizes of $N_{1H}$/$N_{2H}$ and $P_{1}$/$P_{2}$ should satisfy the following:

(8) $${\left(\frac{W}{L}\right)}_{N1,2H}>\frac{V_{H}-V_{\mathrm{tINV}}}{V_{\mathrm{DD}}-V_{\mathrm{NH}}}{\left(\frac{W}{L}\right)}_{P}.$$

As can be seen from (6), the fall time $t_{2}$ can be optimized by altering $V_{\mathrm{tINVH}}$, $I_{0}$ and $R_{P}$ to reduce propagation delay. So, we perform the following arithmetic with $t_{2}$.

(9) $$\frac{\partial t_{2}}{\partial I_{0}}=-\frac{R_{p}C_{A,B}\left(V_{H}-V_{\mathrm{tINVH}}\right)}{I_{0}\left[I_{0}R_{p}-\left(V_{H}-V_{\mathrm{tINVH}}\right)\right]}<0$$

(10) $$\frac{\partial t_{2}}{\partial V_{\mathrm{tINVH}}}=-\frac{R_{P}C_{A,B}}{I_{0}R_{p}-\left(V_{H}-V_{\mathrm{tINVH}}\right)}<0$$

(11) $$\begin{array}{cc} \frac{\partial t_{2}}{\partial R_{p}} & =C_{A,B}ln\left[\frac{I_{0}R_{p}}{I_{0}R_{p}-\left(V_{H}-V_{\mathrm{tINVH}}\right)}\right]\\ & -\frac{C_{A,B}\left(V_{H}-V_{\mathrm{tINVH}}\right)}{I_{0}R_{p}-\left(V_{H}-V_{\mathrm{tINVH}}\right)} \end{array}$$

(12) $$\frac{\partial {}^{2}t_{2}}{\partial R_{p^{2}}}=\frac{C_{A,B}\left(V_{H}-V_{\mathrm{tINVH}}\right)}{R_{p}\left[I_{0}R_{p}-\left(V_{H}-V_{\mathrm{tINVH}}\right)\right]}>0.$$

It can be concluded from (12) that ∂t2∂t2∂RP∂RP is positively correlated with $R_{P}$. If we assume $R_{p}\to \infty$, then ∂t2∂t2∂RP∂RP=0. This means that the maximum value of ∂t2∂t2∂RP∂RP is zero. Thus, the relationship between $t_{2}$ and $R_{P}$ is

(13) $$\frac{\partial t_{2}}{\partial R_{p}}<0.$$

On the other hand, it can also be seen from Figure 5 that increasing the resistance $R_{P}$ can help to accelerate the discharge of the capacitor $C_{A,B}$. Therefore, the fall time $t_{2}$ of $V_{A,B}$ will reduce with the increase of $R_{P}$.

As can be observed from (9), (10) and (13), the fall time $t_{2}$ is negatively correlated with $I_{0}$, $V_{\mathrm{tINVH}}$ and $R_{P}$. In other words, increasing $I_{0}$, $V_{\mathrm{tINVH}}$ and $R_{P}$ can reduce the fall time $t_{2}$. We can use large-sized devices $N_{1H}$/$N_{2H}$ to increase current $I_{0}$, but it will also increase the chip area and power consumption. The threshold voltage $V_{\mathrm{tINVH}}$ can be raised by enhancing the pull-up ability or by mitigating the pull-down strength of inverter $I_{A1}$/$I_{B1}$. The maximum $V_{\mathrm{tINVH}}$ can be set close to $V_{H}$ - $V_{\mathrm{tp}}$. $V_{\mathrm{tp}}$ is the threshold voltage of the PMOS on the high side. Similarly, $t_{2}$ (${t^{{}^{\prime }}}_{2}$ ) will decrease with the increase of $R_{P}$, while minimizing the ratio W/L of $P_{1}$/$P_{2}$ can increase the pull-up resistance $R_{P}$.

When $V_{A}$ ($V_{B}$) falls below the threshold $V_{\mathrm{tINVH}}$ of inverter $I_{A1}$ ($I_{B1}$), voltage $V_{A1}$ ($V_{B1}$) will be pulled up to high after the delay $V_{\mathrm{tINVH}}$ of inverter $I_{A1}$ ($I_{B1}$) Therefore, the delay $t_{3}$ (${t^{{}^{\prime }}}_{3}$) is the transmission time of inverter $I_{A1}$ ($I_{B1}$).

(14) $$t_{3}=t_{3}^{\prime }=t_{d\_\mathrm{INV}}.$$

As $V_{A1}$ and $V_{B1}$ are both high, $V_{\mathrm{CK}2}$ tends to be low after the delay $t_{d\_\mathrm{Nand}}$ of Nand A1. The read-Reg works in transfer-mode, while pre-Reg is in the latch mode. Then the output state of the pre-Reg is transmitted to the output $V_{\mathrm{OH}}$ after the read-Reg delay $t_{d\_\mathrm{Reg}}$. The read-Reg is composed of two Nand gates with a cross-coupled connection. Usually, we set delay $t_{d\_\mathrm{Nand}}$ the same as the inverter delay $t_{d\_\mathrm{INV}}$. Thus, delay $t_{d\_\mathrm{Reg}}$ is twice the delay $t_{d\_\mathrm{INV}}$. As a result, $t_{4}$ and $t_{5}$ are expressed by

(15) $$t_{4}={t^{{}^{\prime }}}_{4}=t_{d\_\mathrm{Nand}}=t_{d\_\mathrm{INV}}$$

(16) t5=t′5=td_Reg=2td_INV.

Thus, the rising propagation delay $t_{r\_\mathrm{pd}}$ from the rising edge of $V_{\mathrm{INL}}$ to $V_{\mathrm{OH}}$ is found by

(17) $$\begin{array}{cc} \; & t_{r\_\mathrm{pd}}=t_{1}+t_{2}+t_{3}+t_{4}+t_{5}\\ = & t_{d\_\mathrm{NH}}+\tau \ln{\left(1-\frac{V_{H}-V_{\mathrm{tINVH}}}{I_{0}R_{p}}\right)}^{-1}+4t_{d\_\mathrm{INV}}. \end{array}$$

Likewise, the fall propagation delay $t_{f\_\mathrm{pd}}$ from the falling edge of $V_{\mathrm{INL}}$ to $V_{\mathrm{OH}}$ is found by

(18) $$\begin{array}{cc} \; & t_{f\_\mathrm{pd}}=t_{1}^{\prime }+t_{2}^{\prime }+t_{3}^{\prime }+t_{4}^{\prime }+t_{5}^{\prime }\\ = & t_{d\_\mathrm{NH}}+\tau \ln{\left(1-\frac{V_{H}-V_{\mathrm{tINVH}}}{I_{0}R_{p}}\right)}^{-1}+5t_{d\_\mathrm{INV}}. \end{array}$$

As can be seen from (17) and (18), the propagation delay consists of the intrinsic delay of HV-NMOS, logic gate delay, and $V_{A}$ ($V_{B}$) fall time. Due to the trade-off between power consumption and area, it is quite limited to decrease the intrinsic delay $t_{d\_\mathrm{NH}}$ of MOSFET and the delay $t_{d\_\mathrm{INV}}$ of the logic gate. As can be seen from (13), reduction of the fall time $t_{2}$ is a feasible solution to reducing the propagation delay of the proposed FHV-LS. The improvements will be introduced in the next section.

## 4. Improvements of Proposed FHV-LS

**Figure 6:**
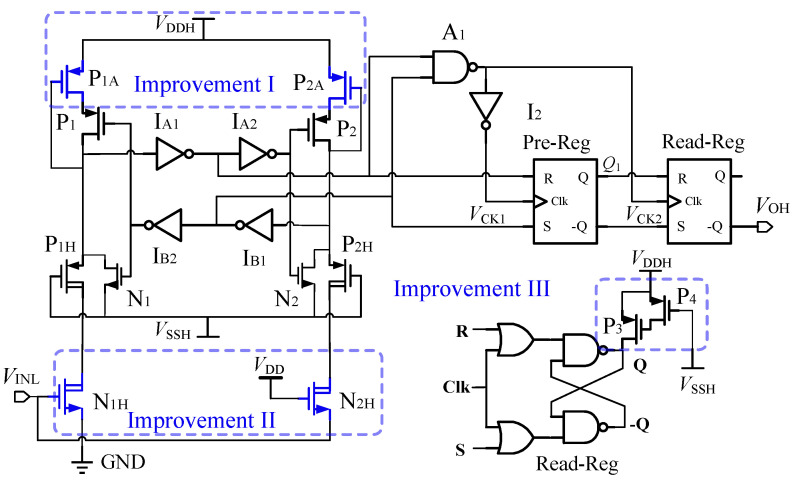
Improvements in propagation delay, symmetry, and startup circuit.

It follows from (13) that the fall time $t_{2}$ is negatively correlated with the on-resistance $R_{P}$ of $P_{1}$/$P_{2}$. During fall time $t_{2}$, the pull-up network $P_{1}$ ($P_{2}$) operating in the linear region has low on-resistance $R_{P}$. It will prolong the discharge time of parasitic capacitance $C_{A}$/$C_{B}$ at $V_{A}$/$V_{B}$ node and also increase the fall time $t_{2}$. Hence, an effective solution to reducing the fall time $t_{2}$ is to increase $R_{P}$. Comparing (17) and (18), the difference between the rising and falling propagation delays is caused by delay $t_{d\_\mathrm{INV}}$ of the low-side inverter. It will induce a deviation between rising and falling propagation delay. With the decrease in propagation delay, the distortion will increase. In this section, three improvements will be performed to optimize the propagation delay, deviation, and start-up of the proposed FHV-LS. Simple improvements can be made to the basic design, as shown in Figure 6. In addition, the device parameters of the improved FHV_LS are summarized in Table 1.

### 4.1. Improvement-I Reduction of Propagation Delay

**Figure 7:**
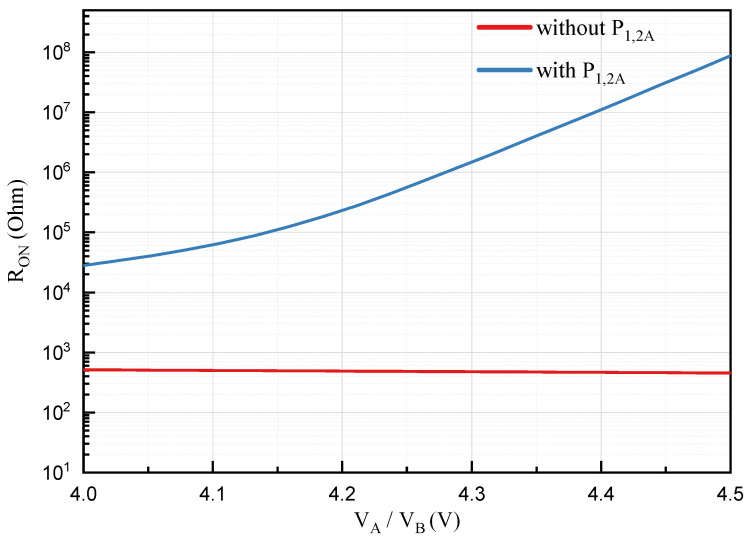
On-resistance of $P_{1}$ ($P_{2}$) with and without $P_{1A}$ ($P_{2A}$).

To decrease the fall time $t_{2}$, the large on-resistance at the drain of $P_{1}$/$P_{2}$ is required. For this purpose, a pull-up network with regulated strength [30,31] composed of $P_{1}$ ($P_{2}$) and $P_{1A}$ ($P_{2A}$) is applied as the improvement-I as depicted in Figure 6. During the fall time $t_{2}$ (${t^{{}^{\prime }}}_{2}$) interval, $P_{1}$ ($P_{2}$) operates in the linear region, and $P_{1A}$ ($P_{2A}$) is in sub-threshold region. In addition, maximizing $V_{\mathrm{tINVH}}$ will be able to reduce the fall time $t_{2}$. Thus, $V_{\mathrm{tINVH}}$ is closed to $V_{\mathrm{DDH}-V_{\mathrm{tp}}}$ by selecting the device size of $I_{A1}$. The on-resistance of $P_{1}$ ($P_{2}$) and $P_{1A}$ ($P_{2A}$) is simulated as illustrated in Figure 7. When $V_{\mathrm{tINVH}}$ is set to 4.2 V, the on-resistance $R_{\mathrm{ON}}$ with $P_{1A}$ ($P_{2A}$) is two orders of magnitude higher than $R_{\mathrm{ON}}$ without $P_{1}$ ($P_{2}$), as $V_{A}$/$V_{B}$ varies between 4.2 V and 5 V, as shown in Figure 7. The resistance $R_{P}$ of the regulated strength pull-up network is higher than the resistance of $P_{1A}$ ($P_{2A}$). Therefore, the fall time $t_{2}$ and propagation delay of proposed FHV-LS will be decreased by the pull-up network with the regulated strength. Due to the reduction in pull-up capacity of the pull-up network with the regulated strength, the rise time of $V_{A,B}$ will increase. Fortunately, the propagation delay of the proposed FHV-LS is independent of the rise time of $V_{A}/V_{B}$ since the pre-storage technique optimizes the transmission path. Compared with traditional resistance enhancement techniques (replace $P_{1A}/P_{2A}$ with passive resistors), the pull-up network with the regulated strength can achieve a better trade-off between propagation delay, power consumption, and chip area.

**Figure 8:**
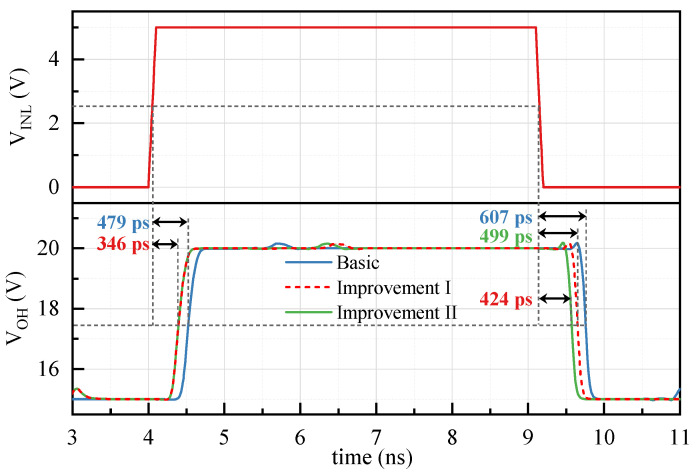
Simulation on the propagation delay with Improvement-I and Improvement-II.

The post-layout simulation with Improvement-I is performed to verify the reduction in propagation delay as depicted in Figure 8. Compared with the basic proposed FHV-LS, the rising propagation delay with Improvement-I decreases from 479 ps to 346 ps, and the fall propagation delay drops from 607 ps to 499 ps. In other word, the average propagation delay is reduced by 22%.

### 4.2. Improvement-II Optimizing the Symmetry of Propagation Delay

The deviation between the rising and falling propagation delays is induced by the transmission delay of inverter I${}_{1}$ on the low side. The common-gate structure is introduced to replace the low-side inverter as Improvement-II [32,33], as shown in Figure 6. The source terminal of $N_{2H}$ is connected to $V_{\mathrm{INL}}$ directly, forming a pseudosymmetry input pair. The input of $N_{1H}$ is the gate, while the input of $N_{2H}$ is the source. $V_{\mathrm{INL}}$ is the common input signal of $N_{1H}$ and $N_{2H}$. The switching processes of $N_{1H}$ and $N_{2H}$ are almost simultaneous. From another perspective, the left and right branches of the proposed FHV-LS are quasi-symmetrical structurally. As a result, the deviation between rising and falling propagation delay will be reduced. However, the transistor $N_{2H}$ exhibits body effect as the source/body is separated. With the same size, the intrinsic delay of $N_{2H}$ is longer than that of $N_{1H}$. Compared with the inverter delay, this difference is very small.

The post-layout simulation with Improvement-II is performed to verify the reduction in deviation between the rising and falling propagation delays in Figure 8. The falling propagation delay decreases from 499 ps to 424 ps, while the rising propagation delay is unchanged. The reduction in falling propagation delay is attributed to the introduction of the quasi-symmetrical input to replace the inverter on the low side. The deviation between rising and falling propagation delay decreases from 36% to 20%. The difference between the rising and falling propagation delays is 75 ps which is caused by the body effect of $N_{2H}$.

### 4.3. Improvement-III Start-Up Circuit

**Figure 9:**
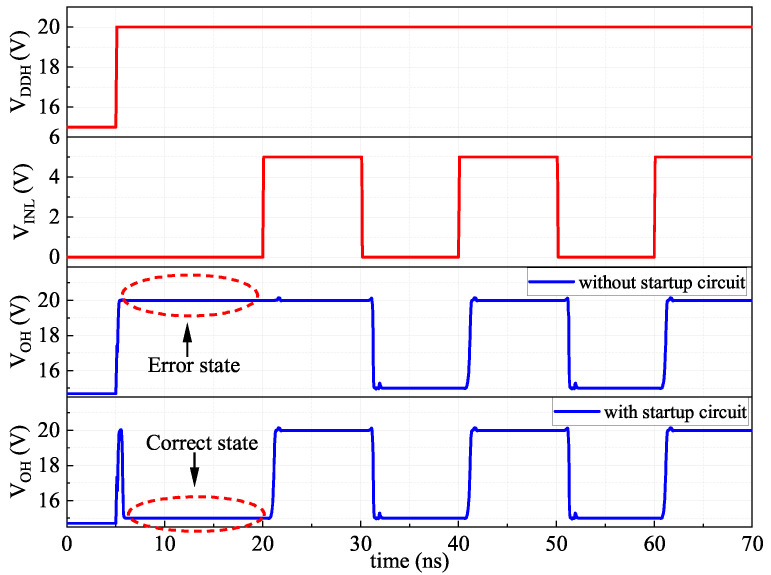
The output state of proposed FHV-LS with and without the start-up circuit during $V_{\mathrm{DDH}}$ power-up.

When $V_{\mathrm{DDH}}$ is powered up, the output of the proposed FHV-LS is a uncertain state due to the read-Reg in latch mode. To overcome this problem, a start-up circuit composed of $P_{3}$/$P_{4}$ is developed to initialize the state of the read-Reg, as shown in Figure 6. During $V_{\mathrm{DDH}}$ power-up, the current of $P_{3}$ pulls the *Q* of read-Reg to high. Consequently, $V_{\mathrm{OH}}$ is low. Then $P_{3}$ is turned off by $P_{4}$. There is no quiescent current consumption on $P_{3}$/$P_{4}$ after $V_{\mathrm{DDH}}$ power-up. The outputs of read-Reg with and without the start-up circuit are simulated in Figure 9. In case of no start-up circuit, the output $V_{\mathrm{OH}}$ of read-Reg is high after $V_{\mathrm{DDH}}$ power-up. It is an error state, which is not in agreement with input $V_{\mathrm{INL}}$. With the start-up circuit, the $V_{\mathrm{OH}}$ is pulled down after $V_{\mathrm{DDH}}$ power-up. As a result, the $V_{\mathrm{OH}}$ follows the input $V_{\mathrm{INL}}$ correctly under any operating conditions.

## 5. Post-Layout Simulation

To comprehensively evaluate the performance of the proposed FHV_LS, post-layout simulations are performed for the propagation delay, power consumption, and stability. The simulation tool used for this work is Cadence Spectre Simulator (Cadence Design Systems, Inc., San Jose, CA, USA), version 6.17. In addition, the design and verification tools for the layout of this wok are Cadence Virtuoso Layout Suite (Cadence Design Systems, Inc., San Jose, CA, USA) and Mentor Graphics Calibre (Mentor Graphics Corporation, Plano, TX, USA), respectively.

### 5.1. Supply Voltage Simulation

**Figure 10:**
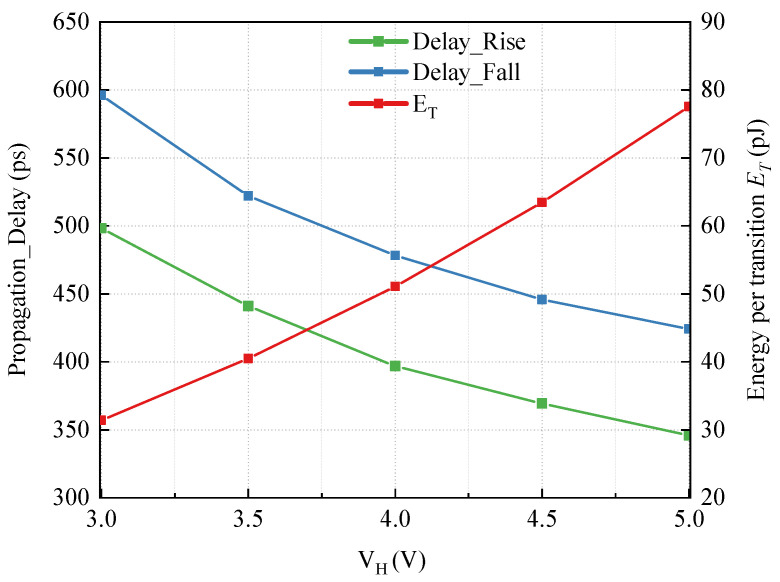
Simulation of the propagation delay and energy per transition ($E_{T}$) versus power supply $V_{H}$.

The average propagation delay and power consumption versus supply voltage $V_{H}$ are exhibited in Figure 10. As $V_{H}$ varies from 3 V to 5 V, the rising propagation delay changes from 498 ps to 346 ps and the falling propagation delay changes from 596 ps to 424 ps, while the $E_{T}$ changes from 31.5 pJ to 77.5 pJ. From Equations (17) and (18), it is known that the propagation delays consist of the delays of logic gates and the fall time of $V_{A,B}$, which are nonlinear with respect to the supply voltage, taking Equations (3) and (4) into condition. Therefore, there is a nonlinear relationship between the propagation delay and supply voltage. On the other hand, the delay of logic gates, as can be seen from Equations (17) and (18), is the major component in the propagation delay. Based on the principle of integrated circuit, the propagation delay of logic gates decreases as the supply voltage increases. As a result, it can be found from Figure 10 that the propagation delays of proposed FHV-LS are negatively correlated with the supply voltage. To achieve low propagation delay, higher supply voltage is required, but power consumption is increased as well. In addition, the rising propagation delay is lower than falling propagation delay since the body effect exists in the input device $N_{2H}$. With Improvement-II, the deviation between rising and falling propagation is lower than 17%.

### 5.2. Process Corners Simulation

**Figure 11:**
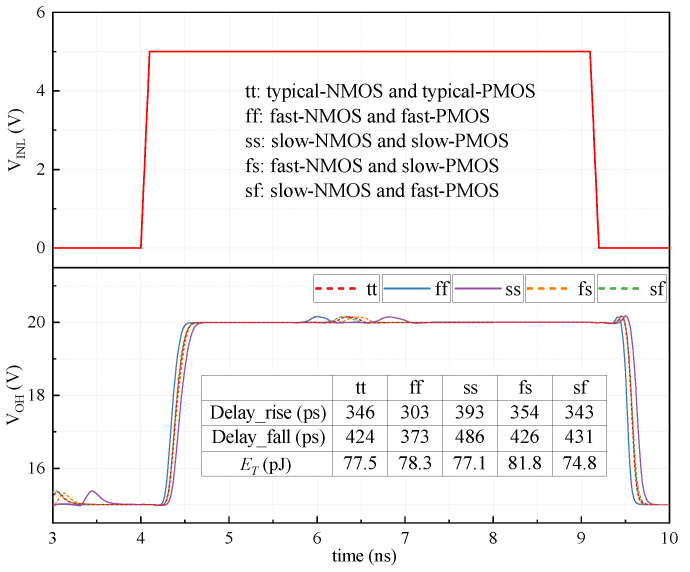
Simulations on the propagation delay and $E_{T}$ at different process corners.

The propagation delay and $E_{T}$ at different process corners are simulated in Figure 11. The rising and falling propagation delay at typical process corner are 346 ps and 424 ps, respectively. Based on the typical process corner, the drift value on propagation delay at ss and ff corners is lower than 15%, and the drift value on propagation delay at fs and sf process corners is lower than 2.5%. As can be seen from Figure 11, the maximum power consumption of $E_{T}$ appears at the fs corner. At this corner, the logic threshold of $I_{A1}$ is lower than that of other corners. As a result, the dynamic power consumption of $I_{A1}$ at the fs corner will increase. Conversely, the dynamic power consumption of $I_{A1}$ at the sf corner will decrease for the higher logic threshold, since the logic thresholds at tt/ff/ss corners are almost unchanged, and they also have similar energy per transition ($E_{T}$).

### 5.3. Temperature Simulation

**Figure 12:**
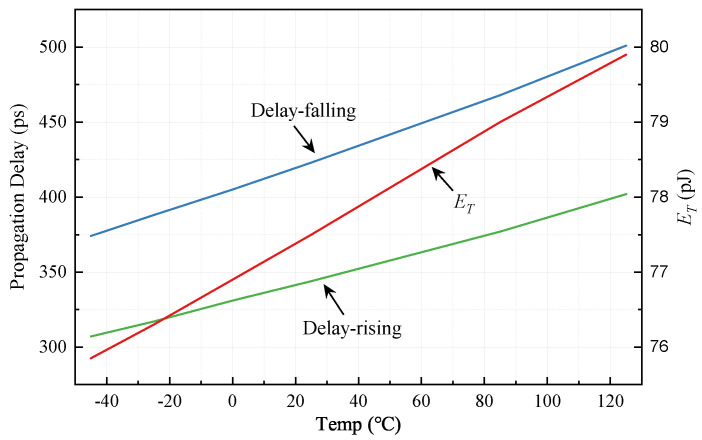
Propagation delay and $E_{T}$ versus temperature simulation.

The simulation of the propagation delay and $E_{T}$ at different temperature are shown in Figure 12. As temperature varies from −40 ${}^{\circ }$C to 125 ${}^{\circ }$C, the rising and falling propagation delays increase from 307 ps to 402 ps and from 374 ps to 501 ps, respectively. Therefore, the temperature coefficients of the rising and falling propagation delay are 0.75 ps/${}^{\circ }$C and 0.56 ps/${}^{\circ }$C, respectively. As can be seen from the figure, $E_{T}$ is positively correlated with the temperature as well. The temperature coefficient of $E_{T}$ is 0.024 pJ/${}^{\circ }$C.

### 5.4. Monte Carlo Simulation

**Figure 13:**
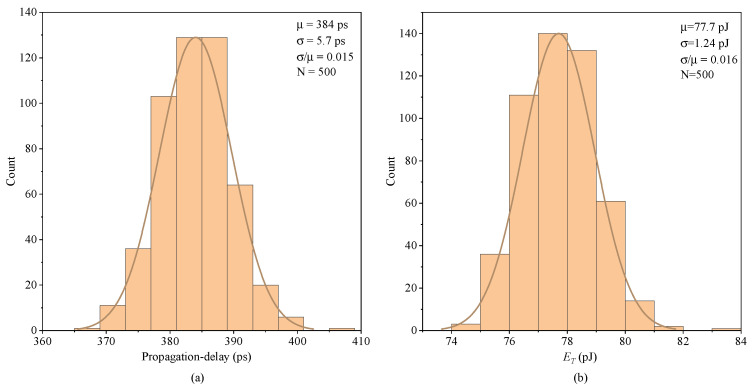
Monte Carlo simulation: (**a**) Average propagation delay, (**b**) $E_{T}$.

To study the variation against the device mismatch and process, 500-point Monte Carlo simulations on the propagation delay and $E_{T}$ of the proposed FHV-LS are carried out in Figure 13. The simulation conditions are $V_{\mathrm{DDH}}$ = 20 V, $V_{\mathrm{SSH}}$ =15 V, $V_{\mathrm{DD}}$ = 5 V, and $f_{\mathrm{VINL}}$ = 1 MHz. With statistical analysis, the means of the propagation delays and $E_{T}$ are 384 ps and 77.7 pJ, respectively. Their normalized standard deviations (σ/μ) are 0.015 and 0.016, respectively. As can be seen from the simulation results, the propagation delay and $E_{T}$ have good stability against process variation and devices mismatch.

### 5.5. Discussion

To reduce the propagation delay, a pull-up network with regulated strength is applied to increase the pull-up resistance. The advantage of Improvement I is that the fall time of $V_{A}/V_{B}$ is reduced. On the one hand, the rise time of $V_{A}/V_{B}$ is increased due to the large pull-up resistance. As a result, the maximum operating frequency should not exceed 100 MHz for the proposed FHV-LS. However, the operating frequency of insulated gate bipolar transistor (IGBT) and SiC power devices are lower than 10 MHz. Therefore, the frequency of 100 MHz is enough for the applications. On the other hand, the immunity for dV/dt of $V_{\mathrm{SSH}}$ is reduced compared with the basic proposed FHV-LS. The maximum immunity for dV/dt of $V_{\mathrm{SSH}}$ is 5 V/ns. Therefore, the proposed FHV-LS is appropriate for the application of half-bridge with low dV/dt or the half-bridge consisting of CMOS structure.

Table 2 summarizes the performance of the proposed LS and previous works. The process, supply voltage, propagation delay, $E_{T}$, area, and figure of merit (FoM and FoM*) are listed in the table. The average propagation delay of the proposed FHV-LS is 384 ps using 0.32 μm HVCMOS technology. In addition, FoM from [16] and FoM* from [20] are 0.06 and 38, respectively. The low propagation delay of this work mainly benefits from the pre-storage technique and the pull-up network with regulated strength. Specifically, the pre-storage technique decreases the number of delay units along the signal transmission path from $V_{\mathrm{INL}}$ to $V_{\mathrm{OH}}$, leading to optimal transmission paths. To reduce the major delay $t_{2}$ in the propagation delay, a pull-up network with regulated strength is employed to increase the pull-up resistance $R_{P}$. Consequently, the proposed FHV-LS features low propagation delay and FoM, as shown in Table 2. In addition, the propagation delay can be optimized according to the analysis of the propagation delay. Based on the operating mechanism of the integrated circuit, the power consumption can be reduced by increasing the propagation delay. In accordance with application requirements, the propagation delay and power consumption of the proposed FHV-LS can be optimized to achieve the trade-off.

## 6. Conclusions

In this paper, a novel FHV-LS with pre-storage technique is proposed to achieve low propagation delay. In the steady state, the input state of FHV-LS is stored in a register. As soon as the input starts switching, the state stored in the register is transmitted to the output of FHV-LS under the control of the CLK generator. Thus, the propagation delay of the proposed FHV-LS is reduced to the sub-nanosecond scale. Since the fall time $t_{2}$ in propagation delay is inversely correlated with the pull-up resistance $R_{P}$, a pull-up network with the regulated strength is used to increase the pull-up resistance $R_{P}$. Therefore, the average propagation delay reduces. To reduce the deviation between rising and falling propagation delay, a pseudosymmetry input pair is introduced to improve the symmetry of the proposed FHV-LS structurally. Moreover, a start-up circuit is designed to initialize the output state of FHV-LS during power-up. Post-layout simulation indicates that the average propagation delay and $E_{T}$ of the proposed FHV-LS are 384 ps and 77.7 pJ at $V_{H}$ = 5 V. Monte Carlo simulation results demonstrate that the proposed FHV-LS has good stability against process variation and devices mismatch.

## Figures and Tables

**Table 1:** Devices parameters of the proposed FHV_LS.

Devices	$N_{1H}/N_{2H}$	$P_{1H}/P_{2H}$	$N_{1}/N_{1}$	$P_{1}/P_{2}/P_{3}/P_{4}$	$I_{A1}/I_{B1}$	$I_{A2}/I_{B2}$	Pre-Reg
				$P_{1A}/P_{2A}$		$A_{1}/I_{2}$	Read-Reg
W/L	40/0.6	24/0.4	10/0.32	10/0.37	N:60/0.32	N:10/0.32	N:10/0.32
(μm/μm)					P:5/0.37	P:10/0.37	P:20/0.37

**Table 2:** Comparison with previous work.

	Process	Voltage	Delay	$E_{T}$	Area	FoM	FoM*	
	μm	V	ns	pJ	μm${}^{2}$			
[15]	0.5	30	1.7	NA	6500	0.11	NA	Measured
	HVCMOS							
[16]	0.5	40	1.7	NA	NA	0.09	NA	Measured
	HVCMOS							
[17]	0.35	20	2.4	24	NA	0.34	67	Measured
	HVCMOS							
[20]	0.18	20	0.37	7.2	4849	0.1	23	Measured
	HVCMOS							
[21]	0.18	50	0.53	30.3	17595	0.06	54	Measured
	HVCMOS							
[25]	0.3	23	1.34	NA	NA	0.19	NA	Measured
	HVCMOS							
[26]	0.18	40	1.4	4.1	7350	0.19	20.4	Measured
	HVCMOS							
[28]	0.18	50	0.5	NA	NA	0.06	NA	Simulated
	BCD							
This work	0.32	20	0.38	77.7	36406	0.06	38	Simulated
	HVCMOS							

FoM from [16]: (Delay)/(Process·Voltage). Unit: ns/(μm·V); FoM* from [20]: (*E_T_*·Delay)/(Process^3^·Voltage). Unit: (pJ·ns)/(μm^3^·V).

## Data Availability

Not applicable.
